# A Type-II Positive Allosteric Modulator of α7 nAChRs Reduces Brain Injury and Improves Neurological Function after Focal Cerebral Ischemia in Rats

**DOI:** 10.1371/journal.pone.0073581

**Published:** 2013-08-09

**Authors:** Fen Sun, Kunlin Jin, Victor V. Uteshev

**Affiliations:** University of North Texas Health Science Center, Department of Pharmacology and Neuroscience, Fort Worth, TX, United States of America; Albany Medical College, United States of America

## Abstract

In the absence of clinically-efficacious therapies for ischemic stroke there is a critical need for development of new therapeutic concepts and approaches for prevention of brain injury secondary to cerebral ischemia. This study tests the hypothesis that administration of PNU-120596, a type-II positive allosteric modulator (PAM-II) of α7 nicotinic acetylcholine receptors (nAChRs), as long as 6 hours after the onset of focal cerebral ischemia significantly reduces brain injury and neurological deficits in an animal model of ischemic stroke. Focal cerebral ischemia was induced by a transient (90 min) middle cerebral artery occlusion (MCAO). Animals were then subdivided into two groups and injected intravenously (i.v.) 6 hours post-MCAO with either 1 mg/kg PNU-120596 (treated group) or vehicle only (untreated group). Measurements of cerebral infarct volumes and neurological behavioral tests were performed 24 hrs post-MCAO. PNU-120596 significantly reduced cerebral infarct volume and improved neurological function as evidenced by the results of Bederson, rolling cylinder and ladder rung walking tests. These results forecast a high therapeutic potential for PAMs-II as effective recruiters and activators of endogenous α7 nAChR-dependent cholinergic pathways to reduce brain injury and improve neurological function after cerebral ischemic stroke.

## Introduction

Clinical management of neuronal damage resulting from ischemic stroke generally involves only palliative treatments. Currently, the only FDA-approved drug therapy for ischemic stroke involves the intravenous use of tissue plasminogen activator (tPA) to dissolve clots [1]. This strategy appears to be effective in ischemic stroke, but only within the first 3 hours after the onset of ischemic stroke [2,3]. This strict limitation reduces the percent of stroke patients eligible for tPA to as low as ~2% [4]. Although in the last two decades substantial efforts have been invested in developing anti-ischemic medicine, these efforts have not resulted in clinically-efficacious therapies for ischemic stroke [5]. These failures highlight the need for development of new therapeutic concepts and approaches for prevention of brain injury secondary to ischemia. Among possible strategies, effective post-stroke treatments with broad therapeutic windows are likely to be the most valuable because of the unexpected nature of stroke. In this search, treatments that are based on recruiting and activating endogenous pathways receive special attention as these approaches are expected to be highly efficacious and cause fewer adverse effects than approaches that utilize exogenous agents [6–8]. To complement these needs, this study evaluates neurological benefits of enhanced activation of α7 nicotinic acetylcholine receptors (nAChRs) by endogenous nicotinic agonists 6 hours after ischemic insult induced by middle cerebral artery occlusion (MCAO) in young adult rats.

There is a substantial body of supportive evidence linking age-, disease- and trauma-related reduction in the expression and function of α7 nAChRs to neurodegenerative, sensorimotor and psychiatric disorders associated with cognitive decline and attention deficits [9–24]. By contrast, activation of α7 nAChRs has been demonstrated to enhance neuronal resistance to ischemia and other insults in *in vivo*, *ex vivo* and *in vitro* experimental models [6,25–39], as well as improved cognitive performance in patients and animal models of neurodegenerative conditions including dementia, schizophrenia, brain trauma and aging [14,26,31,39–61]. An important rationale for the therapeutic use of α7 nAChR agents arises from the fact that α7 nAChRs are ubiquitously expressed throughout the brain [62] including brain regions that are highly vulnerable to ischemia, such as cortex, striatum and hippocampus [63–66]. However, endogenous α7 nAChR agonists (i.e., choline and ACh) have not been regarded as potent therapeutic agents because physiological levels of choline/ACh do not appear to produce therapeutic levels of α7 activation [6]. This limitation has been recently resolved by the use of Type-II positive allosteric modulators (PAMs-II) of α7 nAChRs [6,8,48,67–73]. PAMs-II do not activate α7 nAChRs, but they inhibit desensitization and enhance α7 activation by nicotinic agonists, including endogenous choline and ACh [48,67,68]. Thus, PAMs-II only amplify activation of α7 nAChRs by endogenous nicotinic agonists released naturally as needed [8]. Accordingly, we have recently introduced a novel therapeutic paradigm [6] that converts endogenous choline/ACh into potent therapeutic agents for cerebral ischemia by enhancing activation of α7 nAChRs using PNU-120596, a PAM-II. In our previous proof-of-concept study [6], we have reported that a 3 hour pre-treatment with choline+PNU-120596 significantly delayed anoxic depolarization/injury of hippocampal CA1 pyramidal neurons in the complete oxygen/glucose deprivation model of ischemic stroke in acute hippocampal slices and activation of α7 nAChRs was required; while intravenous administration of PNU-120596 30 min post-ischemia in the MCAO model of ischemic stroke significantly reduced cerebral infarct volume [6]. The present study extends our previous findings and the therapeutic promise of PAMs-II by revealing that PNU-120596 reduces both the focal ischemia-induced cerebral infarct volume and neurological deficits even when administered as long as 6 hours after the ischemic onset. The results of this study further support the potential therapeutic utility of PAMs-II as effective recruiters and activators of endogenous α7-dependent cholinergic pathways to reduce brain injury and improve neurological function secondary to focal cerebral ischemia.

## Materials and Methods

### Ethics Statement

Young adult male Sprague-Dawley (S.-D.) rats (~280 g) were used in experiments. The animal use was in accordance with the Guide for the Care and Use of Laboratory Animals (NIH 865-23, Bethesda, MD), and all experimental protocols were approved by the Institutional Animal Care and Use Committee of University of North Texas Health Science Center at Fort Worth, TX.

### Animals

In total 22 animals were used in this study. Animals were housed 2 per tub in a Tecniplast Green Line IVC Sealsafe PLUS Rat rack on 1/8” corn cob bedding, with Envirodri shredded paper for enrichment. Animals were fed Purina Lab Diet 5LL2, and received filtered water via water bottles. Room lighting was kept below 50 Foot Candles (range of 30-40), and with a timer controlled 12:12 light dark cycle. Room temperature was maintained between 68–72 degrees, with humidity range of 30-70%. Cages were cleaned or changed at least once per week. The housing room contained only rats. The UNTHSC animal facility is AAALAC accredited and follows or exceeds all of the requirements of the Guide for the Care and Use of Laboratory Animals.

### Middle cerebral artery occlusion (MCAO)

Transient (90 min) focal cerebral ischemia was induced using the suture occlusion technique as previously described [74]. Animals (n=22; Charles River, Wilmington, MA) were anesthetized with 4% isoflurane mixed with 67% N_2_O and 29% O_2_ and delivered by a mask. After a midline incision in the neck, the left external carotid artery (ECA) was carefully exposed and dissected. A 19-mm, 4-0 monofilament nylon suture was inserted from the ECA into the left internal carotid artery to occlude the origin of left middle cerebral artery. After 90 min of occlusion, the thread was removed to allow reperfusion. The ECA was ligated, and the wound was closed. Rectal temperature was maintained at ~37° C using a heating pad.

A total of 22 animals were used in this study of which 1 animal from the control group died during the first hours of post-MCAO recovery prior to vehicle injections and another animal from the same control group died after vehicle injection, but prior to behavioral tests. Thus, the mortality rate was ~16.7% in the control group and 0% in the treatment group.

### Drugs

PNU-120596 was obtained from the National Institute of Drug Addiction through the Research Resources Drug Supply Program as well as purchased from Selleck Chemicals (Houston, TX). Other chemicals were purchased from Sigma-Aldrich (St. Louis, MO).

### PNU-120596

In all experiments of this study, 1 mg/kg PNU-120596 was administered via intravenous (i.v.) injections. Similar doses have been used in other studies [6,48,71,73]. To make a 50 mM stock solution (maximal achievable concentration is ~200 mM), PNU-120596 was dissolved in dimethyl sulfoxide (DMSO). The appropriate amounts of the stock solution (i.e., PNU+DMSO) or DMSO alone (i.e., vehicle) were injected as a single bolus. The amount of DMSO injected in each animal did not exceed 0.5 ml/kg.

### Infarct Volume Measurements

Rats (n=10 per group) were anesthetized and euthanized by decapitation 24 hrs after MCAO. Brains were removed and coronal sections (2 mm thickness) immersed in 2% 2,3,5-triphenyltetrazolium chloride (TTC) in saline for 20 min at 37° C, then fixed for 2 hrs in 4% paraformaldehyde [75]. Infarct area, left hemisphere area, and total brain area were measured by a blinded observer using the ImageJ software, and areas were multiplied by the distance between sections to obtain the respective volumes. Infarct volume was calculated as a percentage of the volume of the contralateral hemisphere, as described previously [76].

### Neurobehavioral testing

Rats (n=10 per group) underwent neurobehavioral tests to evaluate functional outcome of treatments with PNU-120596. Animals were trained prior to MCAO (training period: 3 days, 3 trials per day) and deficits were assessed 24 hrs thereafter. The order of testing (Bederson➔cylinder➔ladder rung walking) was always the same to keep the testing conditions identical for all animals. Although it is unlikely that subjecting animals to early tests in the sequence facilitated or inhibited the animal performance in the later tests, we cannot completely rule out a possibility of inter-test interactions.

### Bederson test

Bederson score was used to assess the neurological deficit using a four-level scale [77]: 0, normal; 1, forelimb flexion; 2, decreased resistance to lateral push; 3, circling.

### Cylinder Test

Forelimb use bias was analyzed by observing the rat’s movements over 3-minute intervals in a transparent, 18-cm-wide, 30-cm-high poly(methyl methacrylate) cylinder. A mirror behind the cylinder made it possible to observe and record forelimb movements when the rat was facing away from the examiner. After an episode of rearing and wall exploration, a landing was scored for the first limb to contact the wall or for both limbs if they made simultaneous contact. Percentage use of the impaired limb was calculated.

### Ladder rung walking test

The ladder rung walking test is sensitive for quantifying skilled locomotion. The degree of motor dysfunction after MCAO was measured by counting the number of foot-faults of the impaired limbs per round, as described previously [78]. Baseline and post-operative testing sessions consisted of three traverses across the ladder. An error was scored for any foot slip or misstep. The number of errors of the affected forelimb and hindlimb in each trial was counted. The mean number of errors in three traverses was calculated.

### Statistical Analysis

Statistical significance of differences among groups was defined by the p-value (i.e., * p<0.05; *** p<0.001) using the two-tailed Mann–Whitney U-test. A non-parametric Mann–Whitney U-test was used because this study did not assume any specific underlying distribution (i.e., Gaussian) of data and had a relatively small sample size (n=5-10). We recognize that non-parametric statistics are often less powerful than parametric statistics and thus, more prone to Type-II error (i.e., missing significance when it is present) [79]. However, in this particular study, differences among groups have been found significant in all experiments further supporting our conclusions. The results are presented as mean+S.E.M.

## Results

### PNU-120596 significantly reduces cerebral infarct volume

**Figure 1:**
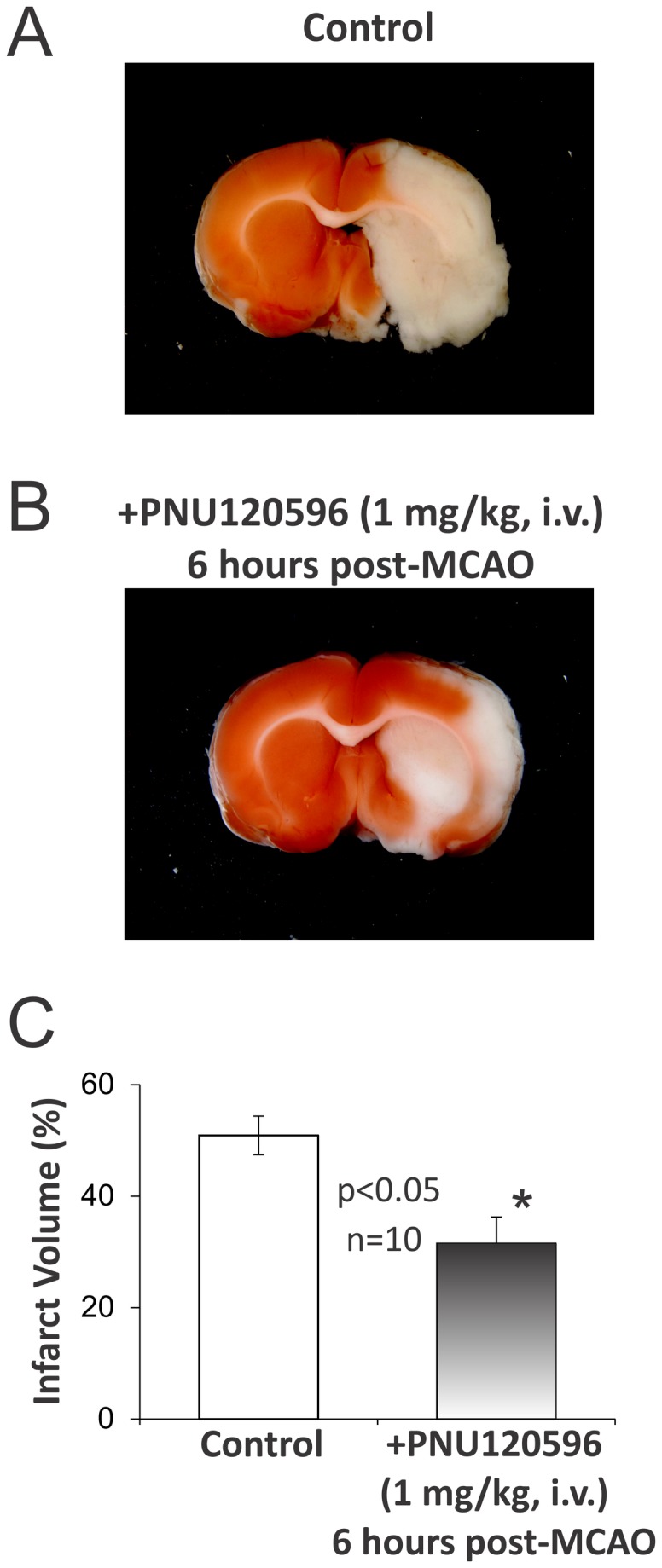
PNU-120596 significantly reduces the size of brain injury induced by focal cerebral ischemia. Focal cerebral ischemia was induced by a transient (90 min) middle cerebral artery occlusion (MCAO). Then, 6 hrs post-MCAO, animals were given i.v. injections of either 1 mg/kg PNU-120596 dissolved in DMSO at 50 mM (i.e., treated group; n=10) or the matched amount of DMSO only (i.e., untreated group; n=10). Typical examples of injured whole-brain coronal sections (2 mm thick) obtained from untreated (i.e., DMSO only) (**A**) and treated (1 mg/kg PNU-120596) (**B**) animals. Treated and untreated animals were anesthetized and euthanized 24 hrs after MCAO (i.e., 18 hrs after PNU-120596 or DMSO injections) and brain sections were prepared for histological analysis. **C**) A summary: MCAO-induced infarct volumes were significantly smaller in treated vs. untreated animals: p=0.0147 (n=10; two-tailed, the Mann–Whitney U-test). The results are presented as mean+S.E.M.

In the group of animals defined as treated, PNU-120596 (1 mg/kg) was administered intravenously (i.v.) 6 hrs post-MCAO and the effects of PNU-120596 on cerebral infarct volume were evaluated 24 hrs post-MCAO using the TTC staining (see Methods). In the matching control group of animals only vehicle (i.e., DMSO) was administered via i.v. injections. Only the left MCA was occluded in each experiment. The results of these experiments demonstrated significant reduction in the infarct volume of treated vs. untreated animals (two-tailed, Mann–Whitney U-test): p=0.0147 (n=10; Figure 1).

### PNU-120596 significantly improves neurological performance post-MCAO

**Figure 2:**
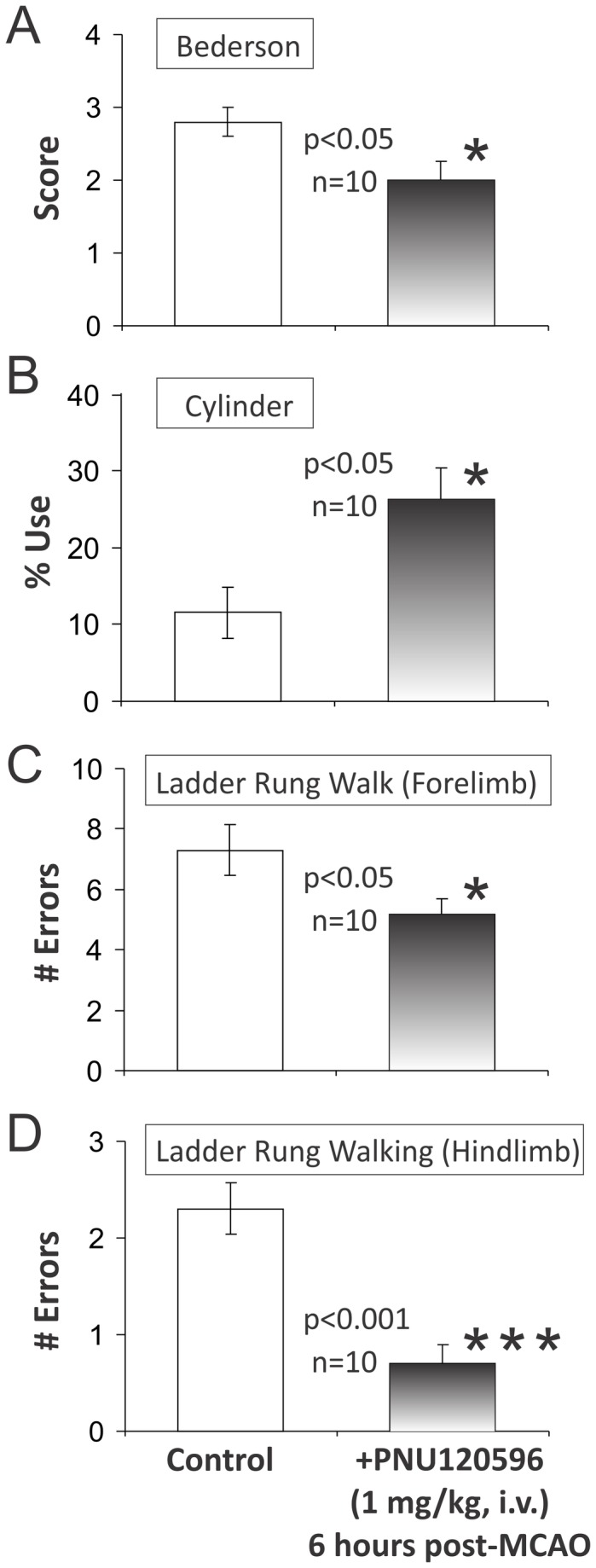
PNU-120596 significantly improves neurological function after focal cerebral ischemia. The same treated (n=10) and untreated (n=10) animals that were used for histological analysis (Figure 1) were subjected to neurological tests 15 min prior to anesthesia/euthanasia and collection of brain sections for histological analysis. PNU-120596 significantly improved neurological function post-MCAO in treated (n=10) vs. untreated (n=10) groups of animals as evidenced by the results of the following tests (two-tailed, the Mann–Whitney U-test): **A**) Bederson, (p=0.0385); **B**) Rolling cylinder, (p=0.0124); **C**) Ladder rung walk, (forelimb), (p=0.0486); and **D**) Ladder rung walk, (hindlimb), (p=0.0007). The results are presented as mean+S.E.M.

The same treated and untreated animals that were used for histological measurements (Figure 1) were used in behavioral experiments 15 min prior to the animal anesthesia/euthanasia and brain tissue collection for histology (i.e., ~24 hrs post-MCAO). PNU-120596 significantly improved neurological function of treated (n=10) vs. untreated (n=10) animals as evidenced by the results of the following behavioral tests (two-tailed, Mann–Whitney U-test): Bederson (p=0.0385; Figure 2A), rolling cylinder (p=0.0124; Figure 2B), ladder rung walking (forelimb) (p=0.0486; Figure 2C) and ladder rung walking (hindlimb) (p=0.0007; Figure 2D). Therefore, the results of these experiments convincingly demonstrate that PNU-120596 produces significant neurological benefits even when it is administered as long as 6 hrs post-MCAO.

## Discussion

The key finding of this study is that PNU-120596, a previously reported highly selective PAM-II of α7 nAChRs, significantly reduces cerebral infarct volume and neurological deficits in the MCAO model of ischemic stroke in rats when the drug is administered as long as 6 hrs post-MCAO. Such a remarkable persistent post-MCAO effectiveness of PNU-120596 invites more comprehensive pre-clinical studies of the PAM-II class of compounds aiming at giving health care providers an effective tool to reducing brain injury and improving neurological function secondary to cerebral ischemic stroke hours after the initial ischemic event. The therapeutic benefits produced by PNU-120596 originate from its ability to convert endogenous agonists of α7 nAChRs (i.e., choline and ACh) into highly potent therapeutic agents [6,48,67,68]. Thus, PAMs-II may create a conceptually novel family of treatments that are based on a novel and substantively different mechanism, i.e., recruiting and activating endogenous α7-dependent cholinergic pathways. Treatments that incorporate endogenous compounds and mechanisms are expected to be highly efficacious and cause fewer adverse effects than treatments that utilize exogenous agents.

These results extend our previous findings that demonstrated a high therapeutic efficacy of PNU-120596 administered intravenously 30 min after focal cerebral ischemia [6]. Intriguingly, infarct volumes measured in animals treated with PNU-120596 30 min (n=5 [6]) and 6 hrs (n=10; this study) post-MCAO were not significantly different (p=0.2404, two-tailed Mann-Whitney test). Similarly, the corresponding infarct volumes measured in untreated animals (i.e., DMSO only) 30 min (n=5 [6]) and 6 hrs (n=10; this study) post-MCAO were also not significantly different (p=0.5921, two-tailed Mann-Whitney test). Therefore, it is likely that the therapeutic efficacy of PNU-120596 extends considerably beyond the 6 hrs post-ischemic delay tested in this study. By contrast, the therapeutic efficacy of donepezil, an inhibitor of ACh hydrolysis, has been reported to cease within the first 2 hrs post-MCAO [80]. Although the reason for these differences is not known, it may be related to the ability of PNU-120596 to inhibit α7 desensitization and thus, generate persistent α7 nAChR-mediated currents in the presence of physiological/endogenous choline [67–69] even though these currents appear to be reduced at physiological temperatures [81]. By inhibiting ACh hydrolysis, donepezil elevates the extracellular levels of ACh (a non-selective agonist of nicotinic and muscarinic AChRs), but does not appear to produce therapeutic levels of nicotinic and muscarinic AChR activation after 1-2 hrs post-ischemia [80].

The therapeutic utility of PAM-II-based strategies is supported by the ubiquitous expression of α7 nAChRs in the brain and especially, in the brain regions that are selectively vulnerable to ischemia, such as cortex, striatum and hippocampus [63–66]. Activation of α7 nAChRs has been shown to enhance neuronal resistance to ischemic and other types of insults [6,31,32,38,39,63,82] as well as improve cognitive performance in patients and animal models of schizophrenia [49,72,73,83], dementia [56,61,84] and traumatic brain injury [39]. Moreover, PNU-120596 has been recently demonstrated to produce robust anti-nociceptive effects by enhancing the potency of endogenous choline for α7 nAChR activation [70,71]. Although choline is a full selective endogenous α7 nAChR agonist, near-physiological levels of choline (i.e., ~20 µM) [12,85–87] are sub-threshold for α7 activation (EC_50_~0.5 mM) [88] and tend to induce α7 desensitization (IC_50_~40 µM) [87]. These limitations can be overcome by the use of PAMs-II, such as PNU-120596. PNU-120596 inhibits α7 desensitization and increases the potency of endogenous choline/ACh for α7 activation producing a weak persistent and tunable activation of α7 nAChRs [67–69] – an activation modality of α7 nAChRs that can benefit neuronal survival as discussed previously [6,27,31,32,38,39,63,82]. Moreover, energy deprivation and cell death/dysfunction can considerably elevate the concentration of choline in the extracellular space [89–91] providing a large source of this endogenous α7 nAChR agonist as has been recently demonstrated by direct measurements of choline/ACh levels in the ischemic core and penumbra in the MCAO model of ischemic stroke in rats [92]. It is intriguing to hypothesize that these elevated levels of choline near the site of injury may robustly enhance neuronal resistance to ischemic injury, while PNU-120596 augments this endogenous therapeutic process [6].

Although the exact cellular and molecular mechanisms of the therapeutic effects of PNU-120596 are not known, α7 nAChR-mediated Ca^2+^-dependent activation of JAK2/AKT-dependent pathways are likely candidates [82,93–95]. These likely mechanisms would be expected to delay mitochondrial dysfunction and thus, PNU-120596-treated neurons may be able to better meet the energy demand of ischemic/hypoglycemic conditions and significantly delay the ultimate failure of the Na^+^/K^+-^ATPase pumps. Such a failure would cause a rapid loss of the neuronal trans-membrane electrochemical gradient leading to transient or terminal anoxic depolarization [6]. It may seem counterintuitive that excitatory currents (i.e., α7 nAChR-mediated) could delay anoxic depolarization and reduce brain injury [6]. However, this concept reflects a common motif in how central neurons respond to insults, i.e., the existence of optimal neuroprotective levels and spatiotemporal patterns of cytosolic Ca^2+^ elevations [8,14,27,32,96–101]. While sub-optimal levels of cytosolic Ca^2+^ are ineffective, excessive Ca^2+^ influx is toxic. By contrast, moderate elevations in cytosolic Ca^2+^ levels, for example, via a K^+^-induced depolarization or weak persistent activation of highly Ca^2+^-permeable α7 nAChRs [102–104] have been shown to protect neurons from injury in a variety of toxicity/insult models [6,27,28,32,33,38,98,105,106]. These therapeutic levels of α7 nAChR activation are consistent with the weak persistent modality of α7 nAChR activation generated by physiological concentrations of choline in the presence of PNU-120596 [67–69].

Moreover, the reported therapeutic efficacy of PNU-120596 may have resulted, at least in part, from enhanced activation of α7 nAChRs expressed in the autonomic neuronal circuitry which may have provided a neurogenic (e.g., adrenergic, nitrergic [107,108]) control over vascular tone and collateral blood circulation. In addition, functional α7 nAChRs are expressed in numerous non-neuronal tissues including glial [109–111] and immune cells [112–114]. Thus, several therapeutic components of α7 nAChR activation in multiple neuronal and non-neuronal tissues may have contributed to the significant therapeutic efficacy of PNU-120596 reported in this and previous *in vivo* studies [6,70–73,114,115]. These potential individual sources of brain protection and their relative contributions to the therapeutic effects of PNU-120596 are not known and present great interest.

One potential limitation of this study is that it does not include experiments with α7 nAChR antagonists (e.g., methyllycaconitine; MLA). Although PNU-120596 is highly selective for α7 nAChRs and to-date non-α7-mediated effects of PNU-120596 have not been reported, there is a slight chance that PNU-120596 activates both α7-dependent and yet unknown, α7-independent pathways. In that unlikely event, the use of highly selective α7 nAChR antagonists would be critical for distinguishing among α7-dependent and α7-independent components of the effects of PNU-120596. However, experiments using MLA *in vivo* may not be straightforward as evidenced from a previous report where the effects of MLA on certain behavioral functions were bell-shaped [116]. Thus, a series of positive and negative controls will need to be conducted using selective α7 agonists (e.g., DMXBA; 3-(2,4-dimethoxybenzylidene)-anabaseine, also known as GTS-21) to determine the effective regimens of MLA as applicable to MCAO. This work has not yet been done in this laboratory.

Another possible limitation is that we have not tested the effects of PNU-120596 on neurological performance of control (sham) animals (i.e., in the absence of MCAO-induced injury). This is because control animals perform these tests nearly flawlessly leaving no room for significant improvement by PNU-120596. However, because of this limitation we cannot exclude the possibility that PNU-120596 is a performance enhancing drug which is also effective in the absence of MCAO-induced injury and thus, the therapeutic efficacy of PNU-120596 post-MCAO may not be directly related to MCAO-induced injury, but extends the performance-enhancing potential of PNU-120596 in the absence of injury.

Certain genetic, age- and trauma-related neurodegenerative, sensorimotor, and psychiatric disorders characterized by cognitive decline and attention deficits (e.g., schizophrenia, dementia and traumatic brain injury) are directly associated with decreased cholinergic tone and a decrease, but not disappearance, of functional α7 nAChRs [10,49,117]. By increasing and partially restoring α7-dependent cholinergic tone, PAMs-II would be expected to improve cognitive function and attention impairments in these patients and animal models [39,49,53,56,61,84]. In this regard, treatments with PNU-120596 or functionally-similar PAMs-II compounds may benefit individuals with ischemic stroke and certain age- and trauma-related cognitive deficits via multiple mechanisms and routes of action.

In conclusion, this study demonstrates a remarkable reduction in the size of cerebral injury and significant improvements in neurological function upon intravenous administration of PNU-120596 as long as 6 hours after the onset of transient focal cerebral ischemia. These results further support the potential therapeutic utility of PAMs-II as effective recruiters and activators of endogenous α7-dependent cholinergic pathways and extend the therapeutic promise of this novel class of compounds.
